# Genome Sequence of the “Indian Bison Type” Biotype of *Mycobacterium avium* subsp. *paratuberculosis* Strain S5

**DOI:** 10.1128/genomeA.00005-13

**Published:** 2013-02-28

**Authors:** Shoor Vir Singh, Naveen Kumar, Shree Narayan Singh, Tapas Bhattacharya, Jagdip Singh Sohal, Pravin Kumar Singh, Ajay Vir Singh, Brajesh Singh, Kundan Kumar Chaubey, Saurabh Gupta, Nitu Sharma, Shailesh Kumar, Gajendra Pal Singh Raghava

**Affiliations:** a Animal Health Division, Central Institute for Research on Goats (CIRG), Makhdoom, Dist. Mathura, Uttar Pradesh, India; b M/S Biovet (P) Ltd., KIADB Industrial Area, III Phase, Malur, Dist Kolar, Karnataka, India; c National JALMA Institute for Leprosy & Other Mycobacterial Diseases (NJIL & OMD), TajGanj, Agra, Uttar Pradesh, India; d Canadian Food Inspection Agency, St. Hyacinthe, Quebec, Canada; e Bioinformatics Centre, CSIR-Institute of Microbial Technology, Chandigarh, India

## Abstract

We report the 4.79-Mb genome sequence of the “Indian Bison Type” biotype of *Mycobacterium avium* subsp. *paratuberculosis* strain S5, isolated from a terminally sick Jamunapari goat at the CIRG (Central Institute for Research on Goats) farm in India. This draft genome will help in studying novelties of this biotype, which is widely distributed in animals and human beings in India.

## GENOME ANNOUNCEMENT

The “Indian Bison Type” biotype of *Mycobacterium avium* subsp. *paratuberculosis* strain S5 was isolated from a terminally sick goat of Jamunapari breed at CIRG (Central Institute for Research on Goats), using the decontamination and culture technique of Merkal et al. (1). This biotype has been recovered from domestic and wild ruminants, rabbits, primates, and human beings in India. This strain has been characterized as a recently evolved *M. avium* subsp. *paratuberculosis* biotype (2, 3). This strain is an antigen source for an indigenous enzyme-linked immunosorbent assay (ELISA) kit and for an indigenous vaccine developed at CIRG for the control of Johne’s disease in animals. Whole-genome sequencing of strain S5 was carried out to explore the genetic organization and genes involved in its physiology, pathogenicity, and immunogenicity.

The genome of strain S5 was sequenced by both Illumina GA IIx, which produced a total of 112,487,226 paired-end reads of length 101 nucleotides (nt), and Ion Torrent technology, which generated a total of 1,151,448 reads of length 5 to 202 nt. We used the next generation sequencing (NGS) quality control (QC) toolkit v2.2.1 (4) to filter the Illumina data for high-quality (HQ) (cutoff read length for HQ = 40%, cutoff quality score = 10) and vector- and adaptor-free reads. A total of 100,506,616 paired-end reads and 5,300,026 single-end reads were obtained after filtering and again were trimmed at the 3′ end (the last 11 bases that have average quality score of <15). We also trimmed all bases of Ion Torrent reads at the 3′ end that had a quality score of <15. We performed reference-assisted genome assembly of filtered data with *M. avium* subsp. *paratuberculosis* strain K10 (GenBank accession no. NC_002944.2) using Velvet v1.2.08 (5). There was a total of 178 contigs of size 4,798,157 nt, with an N_50_ contig length of 58,516 nt; the largest contig assembled measured 199.4 kb and was produced as the draft genome, annotated by RNAmmer 1.2 (6) and the Prokaryotic Genome Annotation Pipeline (PGAAP) (7) of the National Center for Biotechnology Information (NCBI). A total of 4,288 protein-coding sequences (CDSs), 3 rRNAs, and 46 tRNAs were predicted.

Genome annotation by the PGAAP shows that strain S5 contains genes for glycolysis, gluconeogenesis, the pentose phosphate pathway, the tricarboxylic acid cycle, and the glyoxylate cycle. A total of 90 regulator genes were found, which indicates the ability of strain S5 to survive under a wide range of environmental conditions. Large numbers of regulatory genes (~150) were also found in the case of *Mycobacterium avium* subsp. *paratuberculosis* strain K-10 (8). There are 18 oxidoreductases and 18 oxygenases present in the PGAAP annotation, which indicates the role of strain S5 in lipid metabolism and oxidoreduction. A total of 4 serine/threonine protein kinases (STPKs) are also present in the annotation, which are part of the phosphorylation system (9).

Genes, like lipoprotein genes *lpqH* and *lprG*, *pstS*, molecular chaperone gene *dnaK*, chaperonin gene *groEL*, UDP MurNAc hydroxylase gene *namH*, acid phosphatase gene (EC 3.1.3.2), and serine-threonine protein kinase gene *pknG* (EC 2.7.11.1), involved in tuberculosis have been found by mapping all predicted CDSs to the Kyoto Encyclopedia of Genes and Genomes (KEGG) (10) pathways through the KEGG Automatic Annotation Sever (KASS) (11).

### Nucleotide sequence accession numbers.

This Whole Genome Shotgun project has been deposited at DDBJ/EMBL/GenBank under the accession no. ANPD00000000. The version described in this paper is the first version, ANPD01000000.
